# Versatile Microfluidic System for Creating Recirculating Unidirectional Flow for On-Chip Cultures of Barrier Tissues

**DOI:** 10.3390/bioengineering13091076

**Published:** 2026-09-16

**Authors:** Eun-Jin Lee, Longyi Chen, Zachary Krassin, Sabrina Herrmann, Gretchen J. Mahler, Mandy B. Esch

**Affiliations:** 1 Microsystems and Nanotechnology Division, Physical Measurement Laboratory, National Institute of Standards and Technology, Gaithersburg, MD 20899, USA; ejlee09@umd.edu; 2 Department of Chemistry and Biochemistry, College of Computer, Mathematical and Natural Sciences, University of Maryland, College Park, MD 20742, USA; 3 Xinjiang Technical Institute of Physics and Chemistry, Chinese Academy of Sciences, Urumqi 830011, China; chenly@ms.xjb.ac.cn; 4 Department of Biomedical Engineering, Binghamton University, Binghamton, NY 13902, USA; zkrassi1@binghamton.edu (Z.K.); gmahler@binghamton.edu (G.J.M.); 5 University of Saarland, Fraunhofer Institute for Biomedical Engineering, 97070 Wuerzburg, Germany; sabrina.herrmann@ibmt.fraunhofer.de

**Keywords:** microfluidics, pumpless, unidirectional recirculating flow, endothelial barrier

## Abstract

The interaction of chemicals, nanoparticles, and circulating cells with the endothelium depends on the magnitude of the mechanical shear produced by the flow of blood. When simulating those interactions with microphysiological systems (MPSs), it is critical to reproduce those shear conditions faithfully. For example, unidirectional flow of a specific magnitude keeps the endothelium healthy with normal barrier tissue function, while bidirectional flow mimics disease conditions with compromised barrier function. Additionally, in MPS, recirculating fluid may be necessary to retain tissue-derived factors and metabolites. However, existing MPS designs struggle to achieve medium recirculation of small volumes of liquid with precise flow control. Here, we present an MPS design that is highly versatile and overcomes this limitation. We demonstrate how the device can produce a wide range of fluidic flow rates that can accommodate both low shear conditions suitable for tissues that typically are only exposed to interstitial flow and high shear conditions suitable for barrier tissues that experience blood flow. We demonstrate the device’s functionality by culturing human umbilical vein endothelial cells (HUVEC) and confirming their flow-aligned morphology through immunostaining of the adherens junction protein (VE-cadherin) and actin filaments. Furthermore, we present a mathematical model that can be used to calculate operating parameters for culturing any tissue under optimum conditions. We also discuss how the device can be adjusted to recirculate liquid volumes ranging from 100 µL to 5 mL. This versatile system holds promise for commercial applications, including the investigation of expensive compounds that are limited to very small volume samples such as rare cells (e.g., circulating tumor cells) or engineered therapeutic cells with barrier tissues. By offering precise control over a wide range of flow conditions with medium recirculation of small liquid volumes, our device addresses a critical gap in current MPS technology.

## 1. Introduction

Single-organ [1,2] and multi-organ microphysiological systems (MPS) [3] can be used to mimic parts of the human body in experimental settings where human-focused in vitro studies are needed [4,5,6]. For example, the devices can be used to probe for the primary and secondary toxicities of environmental and pharmaceutical compounds in human tissues [7,8,9], as well as a compound’s interactions with barrier tissues such as the endothelium [10,11].

Some MPSs are operated as a one-pass system, where the cell culture medium passes across the tissue and is collected at an outlet [12,13]. Operating one-pass systems can become expensive, especially at the flowrates needed to create physiological conditions in in vitro models of blood vessels that require higher shear forces to establish their barrier function. In addition, in a one-pass system, any compound tested in the system cannot be recirculated unless it is periodically manually transferred from the outlet to the inlet. Recirculating cell culture medium is also advantageous when cells release growth factors that should remain in circulation for the culture to thrive [14].

Recirculating cell culture medium within an MPS can be achieved with pumped, as well as pumpless, gravity-driven systems. Here, it is important to note that pumpless systems generally have smaller dead volumes, keeping the amount of fluid needed to operate the MPS smaller and more physiologically relevant [15,16]. Keeping the recirculating liquid volumes small has the advantage that growth factors produced by the cells remain undiluted, and that any metabolites produced by the cells remain at near-physiological concentrations, making it possible to detect secondary drug toxicity [17,18].

However, typical pumpless systems are limited in terms of the flow rates and flow profiles they can produce and the liquid volumes they can accommodate. Many pumpless systems are operated on rocking platforms using gravity to drive fluidic flow [5]. Gravity-operated devices that are placed on rocker platforms and that are operated without a valving mechanism or flow rectifier produce bidirectional fluidic flow across cell cultures [18,19,20]. A disadvantage of bidirectional flow is that, for example, endothelial cells are sensitive to the direction of flow. It has been shown that human umbilical vein endothelial cells (HUVEC) and lymphatic endothelial cells (HLEC) express elevated levels of inflammation markers such as IL-6, IL-8, and TNF-alpha when they are cultured under bidirectional flow conditions [21,22]. Bidirectional flow can also compromise the endothelial barrier function within 30 min of starting fluidic flow [23]. Even more important is the fact that, when operating microphysiological devices with small amounts of cell culture medium that flows back and forth over a cell culture in a bidirectional flow pattern, small portions of the medium can become trapped within the midsection of the fluidic channel network and become depleted of nutrients and enriched with cellular waste products.

Because of the limitations of bidirectional flow, we and others have developed mechanisms that create unidirectional flow in gravity-driven devices. Those designs include polydimethylsiloxane (PDMS)-based devices such as those by Yang et al. [22] and Wang et al. [24] that were used for endothelial cell culture, as well as a 3D-printed design by Esch et al. [25]. Some of the designs have already been used to operate multi-organ MPS [15,16,26,27,28]. However, many state-of-the-art unidirectional gravity-driven architectures face significant operational trade-offs. Passive-valve systems often require complex multi-layer fabrication [29], suffer from rapid hydrostatic head depletion [30,31], or experience periodic flow interruption [32], whereas fixed-volume designs lack operational flexibility [30]. Consequently, achieving continuous recirculating unidirectional flow across a wide range of working volumes without relying on complex micro-valves remains a key challenge in pumpless MPS.

Here we introduce a versatile and simplified 3D-printed design that can produce stable, unidirectional recirculating flow without complex multi-layer valve integration. We show how, by simply changing the operating parameters, the device can produce a large range of flow rates and a large range of cell culture medium amounts that can be recirculated (100 µL to 5 mL). Flow rates can range from values that are relevant to the culture of endothelial cells [33] generating physiological wall shear stresses, to values that generate low-flow conditions for tissues that typically only experience interstitial flow in vivo. Compared to conventional pumpless platforms—which typically require fixed medium volumes or specialized multi-axis stages—our platform offers superior operational flexibility in working volume and flow consistency, while maintaining low fabrication and assembly overhead (a quantitative comparison with existing gravity-driven MPS platforms is provided in Section 4.5). To demonstrate those approaches, we provide a mathematical model that can be used to calculate differences in flow according to device parameters and changing operational parameters.

The system we developed is easy to use and can be integrated with microfluidic channels made from extracellular matrix. The use of such channels makes it possible to monitor the barrier function of barrier tissues via a microscope and to embed additional cells within the matrix to mimic in vivo conditions more completely. We demonstrate how our device works by culturing HUVECs with and without such an extracellular matrix. We envision that our device will fill a critical gap in MPS technology by enabling high-throughput screening (HTS) of compounds and testing expensive compounds such as engineered cells in small-volume samples.

## 2. Materials and Methods

### 2.1. Microfluidic Device Design

**Figure 1:**
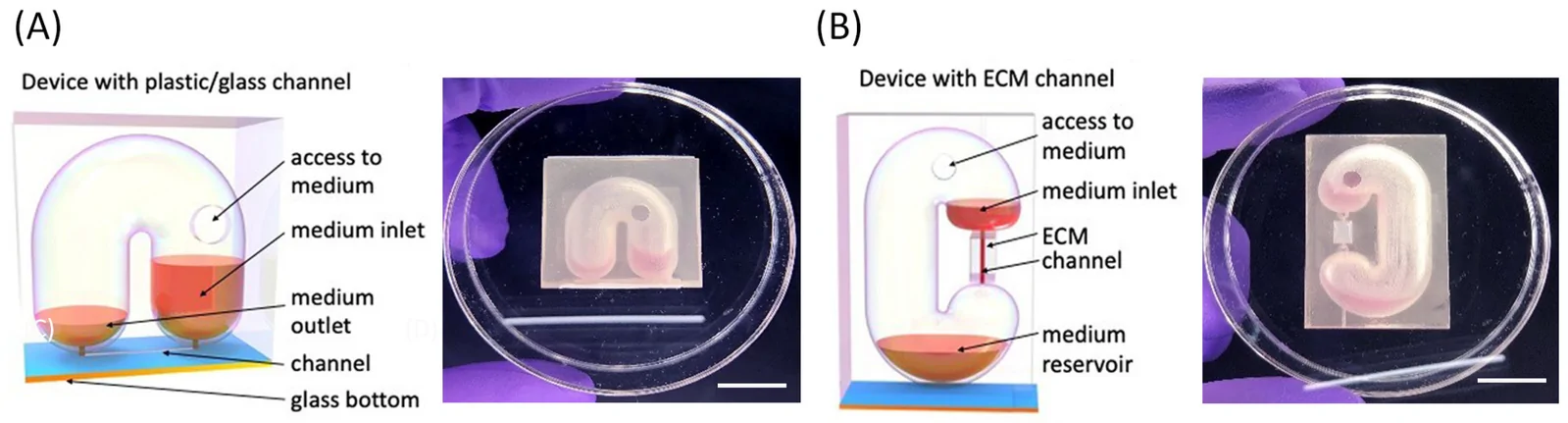
The 3D-printed microfluidic devices with different geometries. (**A**) Schematic drawing of a Design #1 device, featuring a single, smooth U-shaped microfluidic channel designed for surface seeding on the plastic/glass channel floor. Photograph of the fabricated Design #1 device in a petri dish. (**B**) Schematic drawing of a Design #2 device, introducing a distinct central extracellular matrix (ECM) gel channel for true 3D luminal-style cell culture. Photograph of the corresponding fabricated Design #2 device, illustrating the integrated gel channel design (scale bar: 1 cm).

The device we developed consists of three major components: (1) a cell culture channel for the culture of barrier tissue cells such as endothelial cells, (2) an elongated C-shaped tube with round cross section that is 8 mm in diameter and with semicircular endings, and (3) two short, 3 mm long microfluidic channel segments that are 0.5 mm in diameter and that connect the cell culture channel with either a U-shaped tube (Figure 1A, design #1) or a C-shaped tube (Figure 1B, design #2). The two semicircular endings of the tube act as liquid reservoirs that are positioned at different heights, thereby creating a pressure difference between the reservoirs that leads to fluidic flow through the cell culture channel. The longer section of the tube that connects the two reservoirs functions as a conduit that recirculates cell culture medium back into the upper medium reservoir once it has been depleted from there. The shape and size of the reservoirs and fluidic conduit were designed to minimize capillary forces acting on the cell culture medium and thereby prevent unwanted backflow. Specifically, the semicircular endings and round cross section do not provide corners in which cell culture medium can accumulate and travel back to the bottom reservoir.

Design #1, with the U-shaped tube, and Design #2, with the C-shaped tube, are two variations that follow the same design principles but allow for different measurements. Design #1 allowed us to culture HUVEC within conventional plastic/glass channels that were 10 mm long, 0.5 mm wide, and 1 mm deep. The design also had enough space to connect a commercially available flow sensor to the medium reservoirs to measure fluidic flow. Design #2 allowed us to culture HUVEC within a channel (5 mm long, 360 µm in diameter) made from collagen. The dimensions of the microfluidic channels were specifically selected based on fluid dynamics calculations to achieve near-physiological shear stress levels at operating flow rates, while providing an optimal surface area for endothelial cell monolayer formation and high-resolution microscopy imaging.

The physical parameters of the two designs can be altered to change the pressure difference between the two semicircular reservoirs and, with that, achieve a range of flow rates. For example, the tube-shaped channels can be elongated to increase the distance between the medium reservoirs, and the dimensions of the microfluidic cell culture channels, such as length and diameter, can both be adjusted to increase or decrease their hydraulic resistance to fluidic flow.

### 2.2. Fabrication

Device designs with overall outside dimensions of 20 mm × 32 mm × 10 mm were created using 3D computer-aided design software and a high-resolution stereolithography printer (MiiCraft h-50 3D^‡^, Hsinchu City, Taiwan). We utilized company-provided printing material of a proprietary composition (BV007 clear^‡^, CAD works 3D, Concord, ON, Canada) to print the devices. The printed parts were washed with ethanol and cured for 10 s under ultraviolet (UV) light as directed by the supplier. To render the devices biocompatible, we coated the UV-cured devices with a ≈10 µm thick layer of parylene C^‡^ using a vaporization chamber (Labcoter™ 2, PDS 2010^‡^, Indianapolis, IN, USA) loaded with 9 g of parylene C pellets.

We prepared two different versions of the device, each allowing us to perform a different experiment. In design #1 (shown in Figure 1A), the cell culture channel was 3D-printed and closed with a glass cover slip that was glued on using biocompatible adhesive (Torr Seal low vapor pressure epoxy^‡^, Agilent Tech, Santa Clara, CA, USA). In this design, the channel was 10 mm long, 0.5 mm wide, and 1 mm high (Figure 1A). This design enabled us to image the cultured cells using a microscope and fluorescence staining (described in detail in Section 2.9). We also utilized design #1 to connect a flowmeter (stand-alone unit from CorSolutions Inc.^‡^, Ithaca, NY, USA) to the devices to measure fluidic flow rates when operating the devices at various settings. Here, it should be noted that these flow rate measurements do not exactly replicate the flow rates inside the microfluidic channels of our devices we used for HUVEC culture, but they are an approximation and provide evidence of the directionality of the flow.

Design #2 (shown in Figure 1B) contained an opening that allowed us to cast a mixture of collagen around a 28-gauge needle (Figure 1B), as previously described [34]. This procedure produced a round microfluidic channel that was approximately 360 µm in diameter and 5 mm long. Specifically, we allowed 30 µL of collagen mixture to polymerize for 45 min at 37 °C around the needle. The needle was then removed, and the access holes were closed with tape. Design #2 was used to culture cells inside a collagen channel.

### 2.3. Rotating Platform

**Figure 2:**
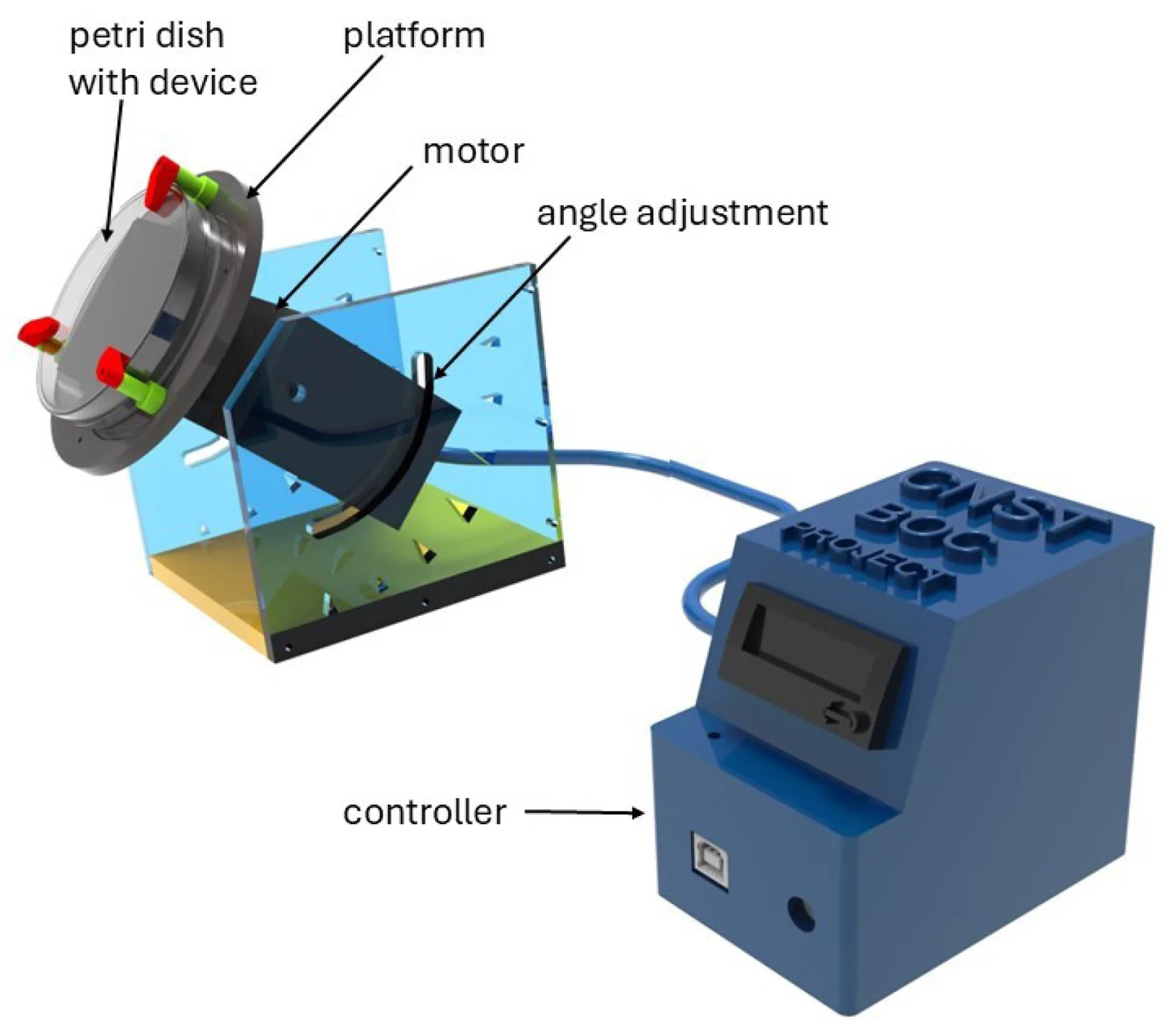
Custom-built rotating platform with adjustable platform angle, adjustable speed, and custom-programmable rotation intervals.

Custom rotation platforms (Figure 2) were fabricated as previously described in detail elsewhere [21]. In short, the platform is assembled from custom 3D-printed components (platform, platform holder, motor housing) and commercially available parts (motor, resistor, and Arduino^‡^, Ivrea, Italy). We controlled the rotation frequency and speed using a simple circuit that combines a power supply, a motor, a MOSFET, a proximity sensor, magnets, and an Arduino microprocessor^‡^ (Ivrea, Italy). The platform angle can be adjusted manually using a screwing mechanism that can be loosened to change the angle and tightened to hold the platform in place. The operating parameters of the rotation platform (rotation speed, frequency, and platform angle) were selected to generate a range of relevant shear stress levels (approximately 0.1 to 7 Pa, or 1 to 70 dyn/cm^2^) [35,36] and ensure recirculating fluid flow.

### 2.4. Contact Angle Measurements

Coating the devices with parylene changed the wettability of the material and, consequently, the contact angle for aqueous solutions, including cell culture media. We measured the contact angle for the cell culture medium before and after coating of the material using a VCA Optima contact angle tool^‡^ (AST Products, Inc., Billerica, MA, USA).

### 2.5. Evaporation

Since the amount of cell culture medium in the devices is small, evaporation can potentially significantly affect flow rates over time. To quantify the amount of water that evaporates over time and to quantify the effects of potential mitigation strategies, we weighed the cell culture medium-loaded devices at various time points with and without mitigation strategies.

### 2.6. Flow Rate Measurements

**Figure 3:**
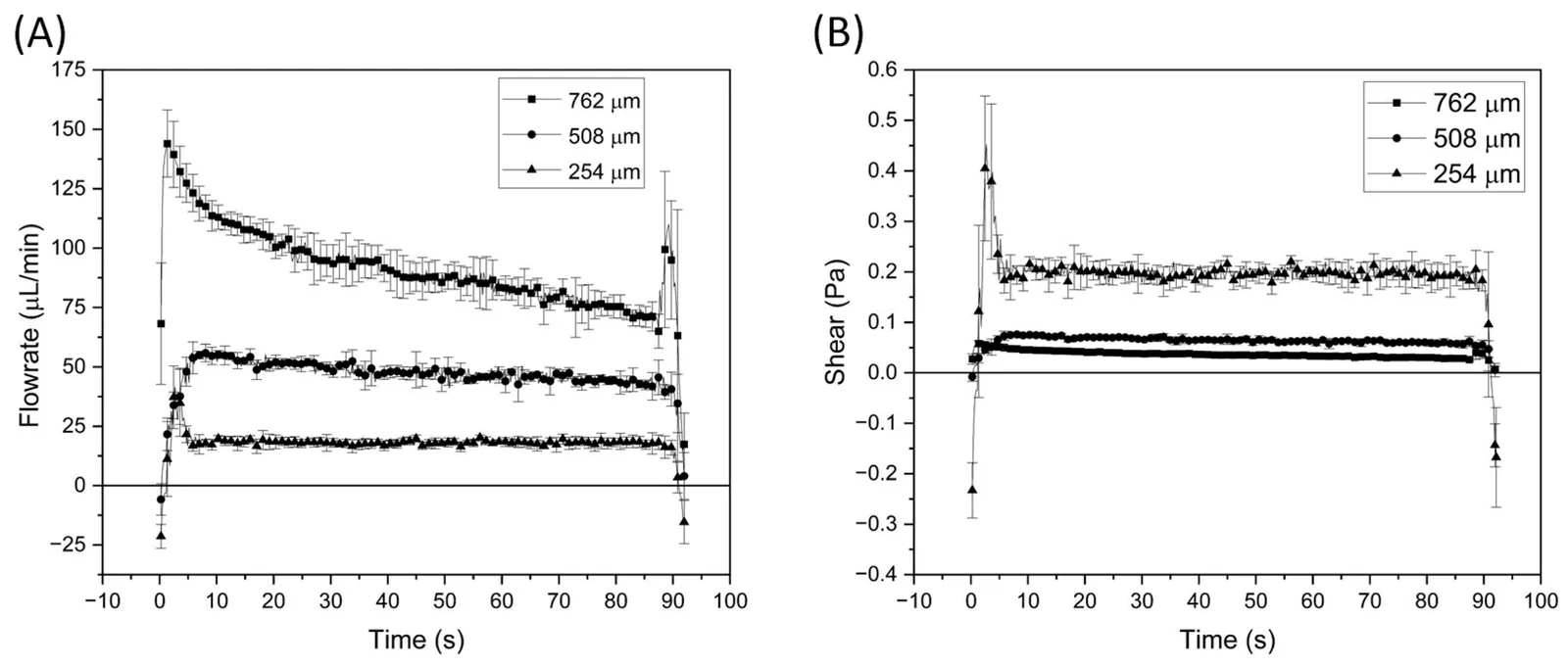
(**A**) Flow rates measured when the device (design 1) is placed on a rotating platform at an angle of 45° and rotated by 360° every 90 s with 762 μm, 508 μm, or 254 μm internal diameter tubing. (**B**) The corresponding calculated wall shear stress. Error bars represent ± the standard deviation of *n* = 3 to *n* = 6 measurements.

**Figure 4:**
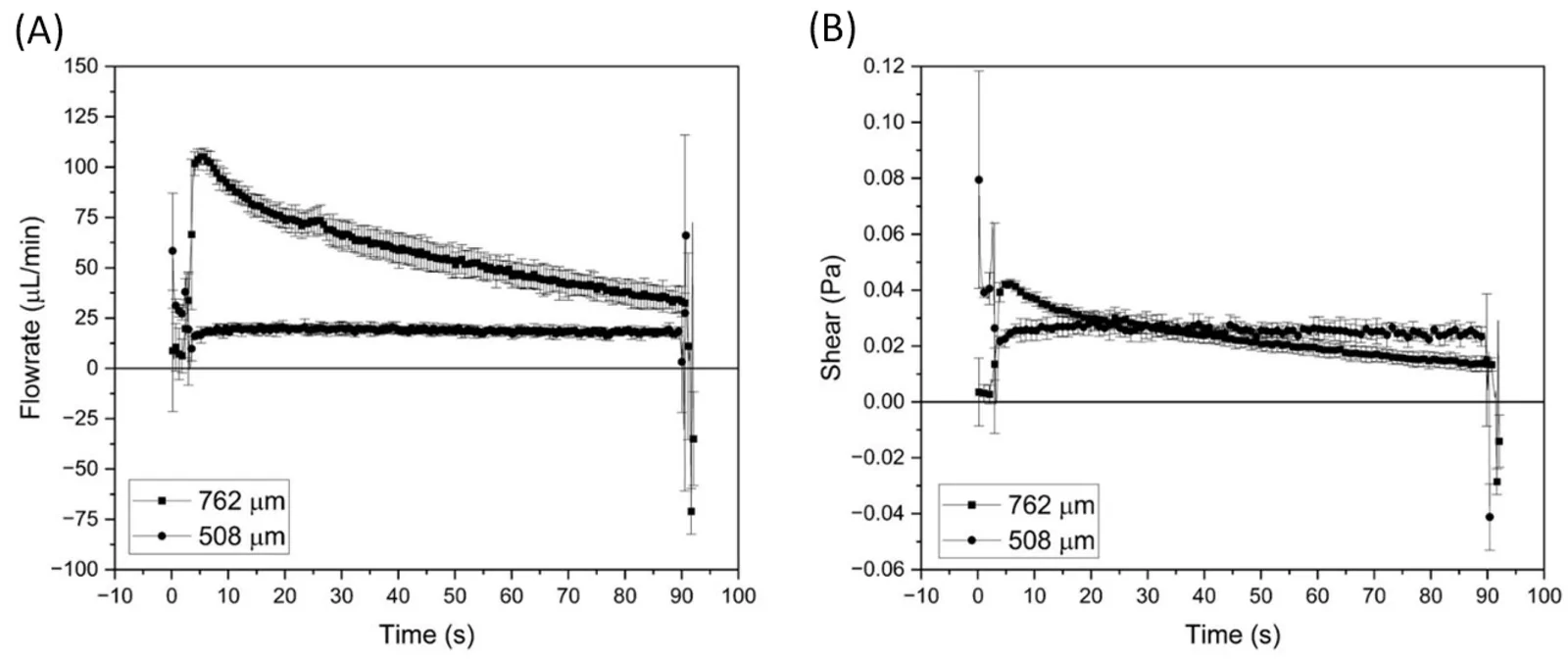
(**A**) Flow rates measured when the device (design 1) is placed on a rocking platform at an angle of 45° and rocked 90 s to the other side for 2 s with 762 μm or 508 μm internal diameter tubing. (**B**) The corresponding calculated wall shear stress. Error bars represent ± the standard deviation of *n* = 3 to *n* = 6 measurements.

Two segments of capillary tubing, 20 mm in length, with inner channel diameters of 508 µm (0.02″) and 762 µm (0.03″), were connected to the devices and made according to the design shown in Figure 1A. Between the two tubing segments, a flow meter (stand-alone unit from CorSolutions Inc.^‡^, Ithaca, NY, USA) was inserted. The devices were then placed on the rocker platform (Infinity Rocker Pro, Next Advance, Inc.^‡^, Troy, NY, USA) that was custom programmed and customized to rock at angles of up to 45°, or on a rotating platform (custom platform as described above), and flow was measured under the following conditions: (1) discontinuous rotation at an angle of 45°, where the device was rotated 360° every 90 s (the rotating itself lasted 2 s), and (2) discontinuous rocking where the device sat at an angle of 45° for 90 s (to flow medium over the cell culture) and at −45° for 2 s (for reloading the medium to the inlet). Here, we used a rocker platform that we modified to accommodate 45° rocking angles, and that was custom programmed to accommodate the 90 s/2 s rocking pattern. Flow rates from 3 to 6 flow cycles (*n* = 3 to 6) were averaged and plotted with standard deviations (Figure 3 and Figure 4).

Shear rates were calculated using the equation for round channel profiles, where Q is the flow rate, η the dynamic viscosity of cell culture medium, and r the radial distance from the center line of the channel [25,37]:

τ = 4 ηQ/r^3^π (1)

### 2.7. Cell Culture

HUVECs, pooled (Lonza C2519A^‡^, Walkersville, MD, USA), were cultured using EBM-2 Endothelial Cell Growth Basal Medium-2 (Lonza CC-3156^‡^, Walkersville, MD, USA) supplemented with EGM-2 MV Microvascular Endothelial SingleQuots Kit (Lonza CC-4147^‡^, Walkersville, MD, USA). The culture medium was exchanged every 48 h. Primary HUVECs (passages 2–6) were maintained in tissue culture flasks and subcultured upon reaching 80–90% confluency, as determined by visual inspection of multiple random fields of view under inverted phase-contrast microscopy (4–10× magnification), ensuring that the cells remained in the log phase of growth prior to device seeding.

To subculture the cells, they were washed with phosphate-buffered saline (PBS) and then exposed to a solution containing a volume fraction of 0.25% trypsin-ethylenediaminetra acetic acid (EDTA). The cells were then collected and spun down at 125 rad/s (≈1200 rpm) and replated in cell culture flasks with 5 mL of fresh culture medium.

Alternatively, for seeding into the devices, the supernatant was removed, and the cell pellet was dissolved in 200 µL of fresh cell culture medium. The cell concentration was adjusted so that it was no lower than 10^6^/mL (We used HUVEC of passage 7 or lower).

### 2.8. Device Setup and Operation

Devices were sterilized by flowing an aqueous solution containing a volume fraction of 70% ethanol through them for at least 15 min. The ethanol-containing solution was then slowly substituted with PBS. PBS was then substituted with PBS containing poly-D-lysine at a concentration of 10 µg/mL. This solution was allowed to sit for 15 min and was then exchanged with PBS containing fibronectin at a concentration of 10 µg/mL. The fibronectin solution was allowed to coat the walls of the device for 60 min and was then replaced with cell culture medium.

Harvested HUVECs of passage number 7 or lower were seeded into the channels of the devices by adding 40 µL of the cell suspension (containing cells at a concentration of about 10^6^/mL) to the channel inlet. The cells were allowed to attach for 60 min in a humidified carbon dioxide (5% volume fraction of CO_2_ in air) incubator at 37 °C. Fresh cell culture medium (200 µL) was then placed into the devices, and the devices were placed on a rotating platform and fluidic flow was started. Once loaded with attached cells, we operated the devices with discontinuous rotation at an angle of 45°, where the device was rotated 360° every 90 s. The cell culture medium was removed and replaced with new cell culture medium every 24 h. Imaging and experiments were conducted after 72 h of culture under fluidic flow. We conducted four technical replicates for each of the five independent biological experiments (*n* = 5).

### 2.9. Immunostaining

The cells were fixed for 15 min with an aqueous solution containing a 2% volume fraction of paraformaldehyde at room temperature. The cultures were then washed with cold PBS containing a volume fraction of 1% bovine serum albumin (BSA, 1% PBSA) and then permeabilized with Triton X-100^‡^ (Midland, MI, USA) in 1% PBSA containing a 0.1% volume fraction of Triton X100 and a 1% volume fraction of BSA for 5 min at room temperature. To visualize vascular endothelial–cadherin (VE-cadherin) and actin fibers, the cells were immune-stained with anti-vascular endothelial (VE)-cadherin antibodies conjugated to Alexa 647 (red, 1:50) and Alexa Fluor 488 phalloidin (green, 1:200) for 2 h on a rotating platform in the dark and washed three times with cold PBS containing a 1% volume fraction of BSA and then rinsed with PBS. Finally, the nuclei were stained with 4′, 6-diamidino-2-phenylindole (DAPI) (blue, 1:1000, emitting light at 461 nm) for 5 min. Cells were then imaged with a fluorescence microscope.

### 2.10. F-Actin Angle Measurement

The angle distribution of HUVEC F-actin was analyzed using the Directionality plugin in Fiji (ImageJ^‡^ 1.54p, NIH, Bethesda, MD, USA) via the Fourier components method [22]. For both HUVECs cultured under unidirectional flow and the static condition, three immunofluorescence images were obtained per channel and per experiment. Each image captured all cells across the entire width of the channel. From each of the three independent experiments per condition, one image was selected at random to measure the F-actin orientation angles.

### 2.11. Barrier Measurements

We used a 10 kDa dextran (labelled with Texas Red) at a concentration of 12.5 μg/mL in medium to assess the barrier function of the endothelium, as previously described [34,38,39]. Diffusion of dextran across the endothelium was imaged in real-time with a fluorescence microscope at 10× magnification. An image time sequence was analyzed by taking the mean intensity over regions next to the cell layer in successive images. The time derivative of the intensity was determined by linear regression for each region. The time derivative of the intensity, the mean intensity (I), and the capillary radius (r) were used to determine the diffusive permeability coefficient (P_d_) by the equation P_d_ = *dI*/*dt* × *r*/2*I*. The uncertainty was determined by the standard deviation of the measured permeability at the different locations.

The positive negative control for full barrier function was a PDMS channel without cells, and the positive control for compromised barrier function was a HUVEC-lined collagen channel that was treated with interleukin 1β (IL1β) at a concentration of 10 ng/mL for 3 days prior to measurements.

### 2.12. Mathematical Model

We developed a mathematical model to estimate changes in the flow rate when changing the device design parameters (channel dimensions or reservoir tube dimensions) or the operational parameters (platform angle). Calculations for inner-diameter tubing of 254 µm, 508 µm, and 762 µm were performed for discontinuous device rotation at several angles.

The pressure drop (ΔP_grv_) driving the fluid through the microfluidic channel is determined by the vertical height difference (H) between the medium levels in the upper and lower reservoirs, governed by the hydrostatic law [40]. This parameter depends on the microfluidic channel length, the amount of cell culture medium being used, and most importantly, the device tilting angle q. Specifically,

ΔP_grv_ = ρgH(q,t) (2)

where ρ is the density of the cell culture medium, and g is gravitational acceleration. Capillarity introduces flow resistance in the form of the pressure ΔP_cap_, acting opposite to the flow, and depending on q. Platform inclination modifies the geometry of the contact region between the cell culture medium and the microfluidic channel, nearing an elliptical form for large amounts in culture media, and changing to a semi-oval form at lower volumes and large tilting angles. This results in a capillarity contact angle f that depends on the tilting angle and the point of measurement at the boundary. In the present study, we approximate the effect of the capillarity force for low volume concentrations as follows

(3) $$\Delta P_{\mathrm{cap}}=\frac{2\sigma \cos\left(\phi \right)}{H\left(\theta \right)\cot\left[\theta \right]+R\csc\left[\theta \right]}$$

Note that, for an untilted channel (*θ* = 90∘), Equation (3) coincides with standard capillary pressure formulations. The net driving pressure is ΔP_grv_ − ΔP_cap_. The hydraulic resistance (Rh) of the microfluidic channel segment is determined by the channel’s dimensions (R, radius, L, length), and the fluid viscosity (µ):

Rh = 8 µL/πR^4^ (4)

The total resistance to flow (R_t_) is determined by a combination of the hydraulic resistances of the microfluidic channel and the resistance the reservoir provides.

R_t_ = R_h,channel_ + R_h,reservoir_ (5)

where R_h,reservoir_ depends on the reservoir diameter, the cell culture medium contact angle with the device surface, and the height of the medium in the reservoir. In this study, we omit the reservoir’s resistance since we expect it to be significantly smaller than the channel’s resistance.

For the channel we are considering here, the resulting flow rate is given by by the q-dependent total pressure and the hydraulic resistances [40]:

ΔQ = (ΔP_grv_ − ΔP_cap_)/R_t_ (6)

In this form, we numerically find H(q) for a fixed medium volume V and inclination q, as a function of time, by solving the equation:

(7) $$\frac{dV}{dt}=-\Delta Q$$

### 2.13. Statistical Analysis

Data are presented as mean ± standard deviation (mean ± SD) from at least three independent experiments (*n* ≥ 3). Statistical comparisons between two groups were performed using a two-tailed Student’s *t*-test, while comparisons across multiple time points were analyzed using a one-way analysis of variance (ANOVA). A *p* value of <0.05 was considered statistically significant. Statistical analyses were performed using GraphPad Prism (version 10.4.1).

## 3. Results

### 3.1. Flow Rates and Flow Patterns

To determine the fluidic flow rates that device #1 can generate, we operated it on both a rocker platform and a rotating platform. The rotating platform was set to a 45° angle and programmed to rotate the device by 360° every 90 s. The rotation took about 2 s, and during the rotation, the cell culture medium travelled along the big reservoir channel from the outlet back into the inlet. The rocking platform was set to stay in one position for 90 s, rock to the opposite position, remain there for 2 s, and then rock back to the first position. The device was placed on the platform in a 45° tilted position, so that medium could flow back into the inlet. Figure 3 and Figure 4 show the measured fluidic flow rates and the resulting calculated wall shear stresses. Among the operating conditions we tested, using the rotating platforms at angles of 45° resulted in the highest flow rates (up to 144 ± 14.1 µL/min at the beginning of a cycle) when using 762 µm tubing and the highest wall shear stress (up to 0.45 ± 0.11 Pa at the beginning of the cycle) when using 254 µm tubing. Predictably, the flow rates generated on the rocking platform were slightly lower because the devices were tilted sideways to allow for the unidirectional flow pattern, thereby decreasing the pressure difference between the two reservoirs. The highest flow rate was 105.3 ± 4.1 µL/min with 762 µm tubing at the beginning of a cycle, and the highest wall shear achieved was 0.04 ± 0.01 Pa with 762 µm tubing at the beginning of the cycle. Operating the devices on both platforms generated the highest flows at the beginning of the cycle, with the flow slowly decreasing over time for high-diameter tubing (762 µm) and settling to stable values for smaller-diameter tubing (≈45 µL/min for 508 µm, and ≈18 µL/min for 254 µm on the rotating platform, and ≈19 µL/min for 508 µm on the rocking platform).

Notably, with both rocking and rotating platforms, the generated fluidic flow was positive (flowing in the positive direction) for most of the operation cycle time (92 s). The flow dipped below zero (indicating backward flow) for 1 s to 2 s during the change of device position on both platforms. The magnitude of this reverse flow was seen with tubing of smaller diameters (254 µm and 508 µm) but not with tubing of larger diameters (762 µm) on the rotating platform (Figure 2).

While the overall flow rates achieved on the rocking platform were lower than those achieved on the rotating platform, operating the devices on a rocker platform was more successful at creating unidirectional flow without negative reverse flow. Although flow rates were measured using an inline external flow sensor connected via tubing, the volumetric flow rate passing through the microfluidic channel directly corresponds to these measured values based on the fluid continuity equation for incompressible liquids, serving as the basis for calculating the average wall shear stresses within the cell culture channel.

### 3.2. Flow Calculations

**Figure 5:**
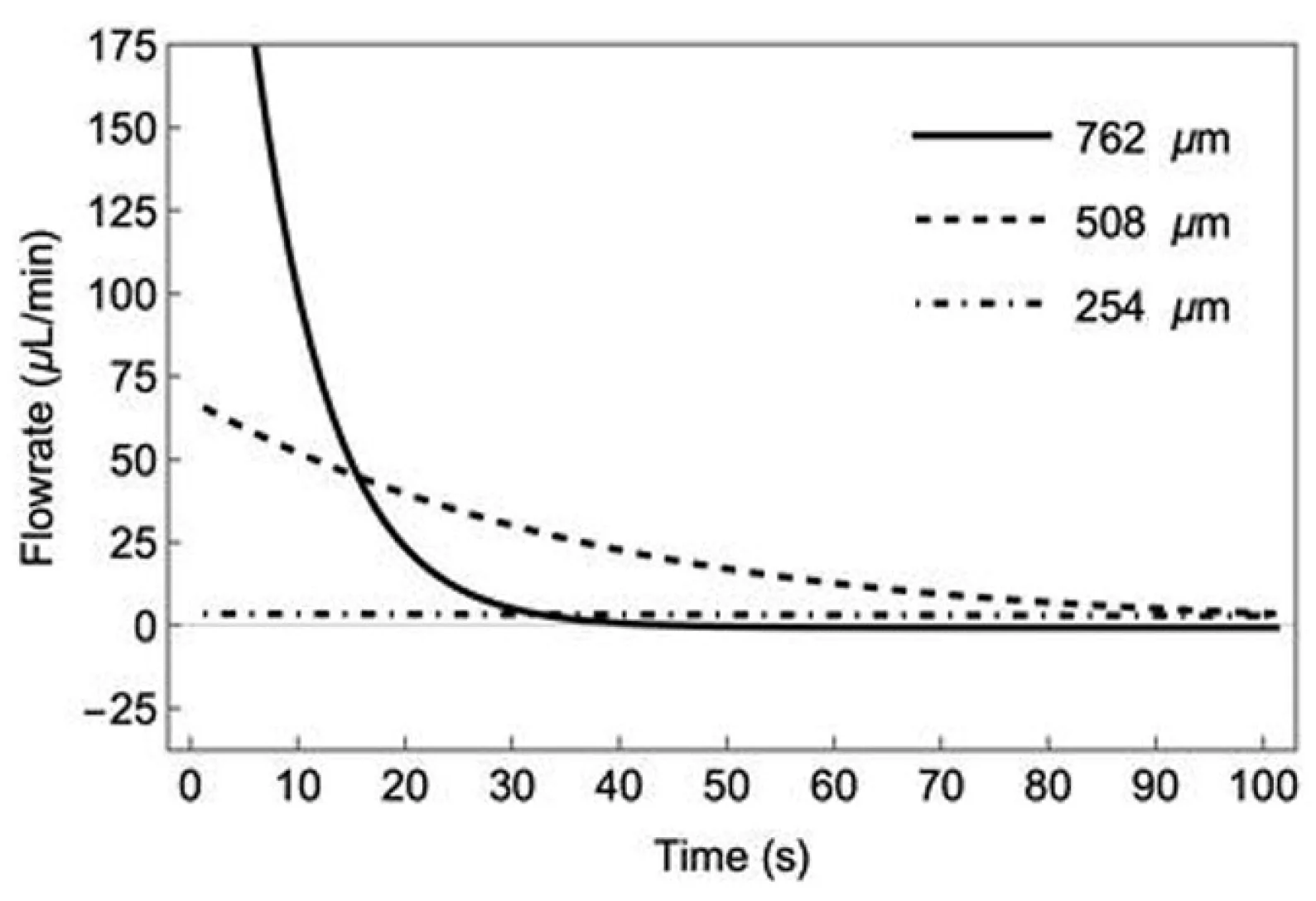
Calculated flow rates for devices (device 1) with 254 μm, 508 μm, and 762 μm internal diameter tubing when they are placed at an angle of 45° and rotated at 90 s intervals.

The overall flow patterns observed on the rotating platform were matched by calculated flow rates for small-diameter flow channels (254 µm and 508 µm) (Figure 5). Similar to the experimental data, our model generates fast flow at the beginning of each cycle, followed by stabilized flow after a few seconds. The model included capillary interactions between the liquid and the device reservoir walls. Those forces become significant for larger diameter channels (762 µm) where hydraulic resistances inside the channel segment decline. For larger diameter channels, our model does not replicate experimental flow rates, likely due to the complex dependence on the geometry of the surface–liquid interaction region in both the inlet and outlet reservoirs. The surface–liquid interactions change significantly for the different angles of device operation, with 90° providing a circular circumference, and angles in the inlets/outlets producing an oval circumference or an oval segment at the interface between the liquid and the device walls. While our model predicts flow rates for devices with channels below 500 µm, further work is needed on the capillary forces in tilted devices to create meaningful predictions for devices with larger diameter channels and angled devices.

### 3.3. Contact Angle and Evaporation

Coating the devices with parylene changed the contact angle for the cell culture medium from 60.0° ± 8.0° to 48.0° ± 2.7°, indicating increased device wettability. Evaporation of water from the devices over a 24 h period was 7.1% ± 1.8% in 3D-printed/glass devices and 12.3 ± 4.0% in devices operated with collagen. Since glass-covered devices are closed except for a small access hole for pipetting medium (2 mm in diameter), evaporation was limited to those values by adding a wetted sponge to the petri dish that kept the humidity high. The devices that are operated with collagen have an extra opening that led to slightly higher evaporation rates. Replacing the cell culture medium every 24 h allowed us to reset the medium level to the original values (in addition to removing cellular waste products and replacing nutrients).

### 3.4. Cell Viability and Alignment

**Figure 6:**
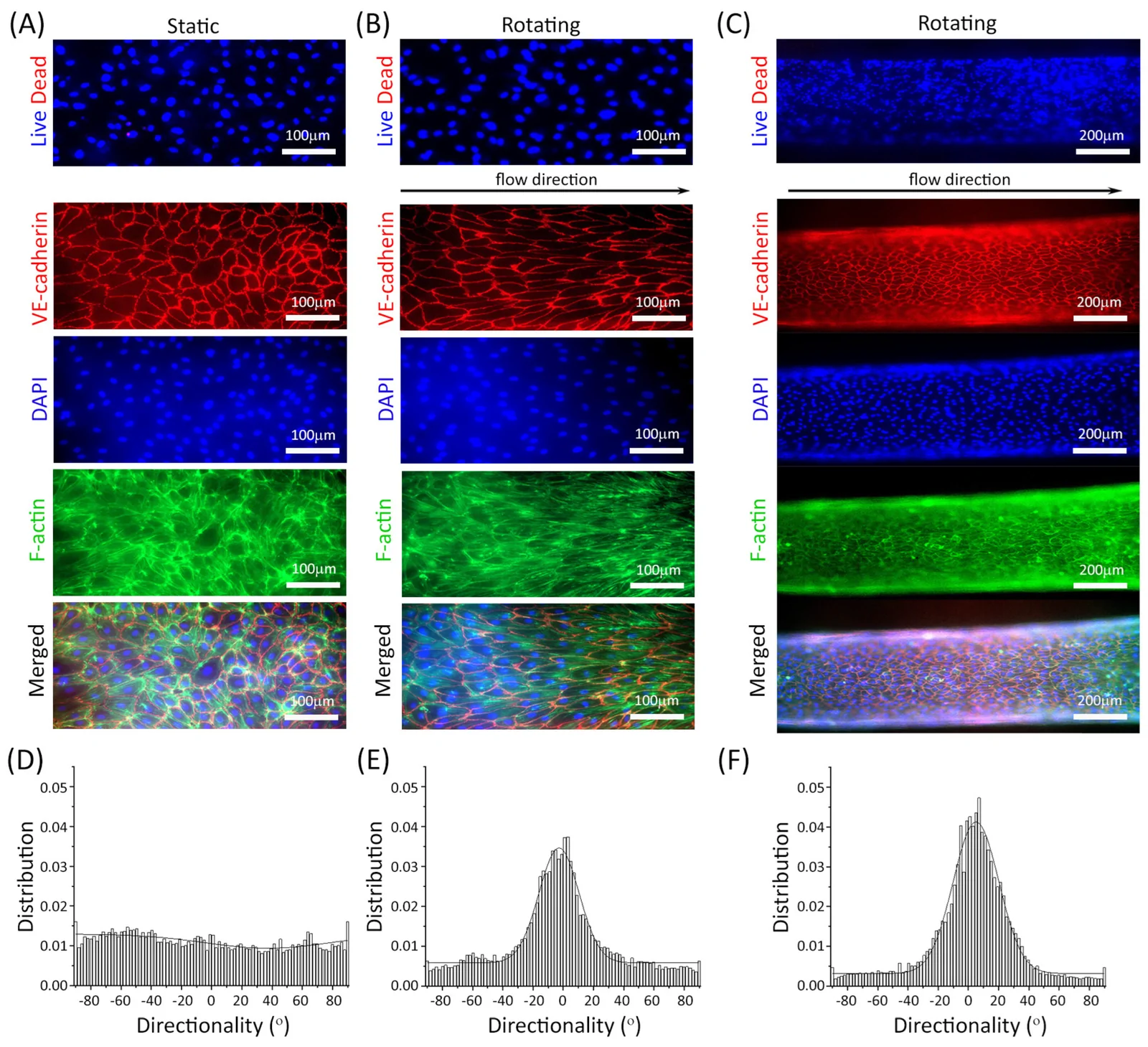
Representative images of human umbilical vein endothelial cells (HUVEC) cultured in static and rotating fluidic devices. The devices were operated on a rotating platform at an angle of 45° with 90 s rotating intervals. (**A**) HUVEC cultured in 3D-printed channels with glass bottoms under static conditions, (**B**) HUVEC cultured in 3D-printed channels with glass bottoms under flow conditions, and (**C**) HUVEC cultured in collagen channels under flow. Note that the collagen channels are round, and images were taken with a focus on the channel center. Therefore, the outer edges of the channels are out of focus. Top Panel: representative images of live (blue) and dead (red) cells. Cell viability on day 1 (97.6 ± 2.9%) and day 3 (95.1 ± 2.9%) showed no significant difference (*p* > 0.05). Bottom Panel: immunofluorescence images showing VE-cadherin (red), DAPI (blue), and F-actin (green). (**D**–**F**) show the alignment of cell bodies obtained under the three culture conditions. Cell alignment distribution parameters (Gaussian fit standard deviation σ = 26° for Design 1, σ = 30° for device 2 on day 3) represent data from *n* = 3 independent experiments.

To determine optimum cell culture conditions for endothelial cells, we cultured HUVEC in both device designs and operated them under flow conditions that created physiologically relevant magnitudes of fluidic shear stress on the endothelial cell layer. Figure 6A,B show HUVEC cultured in 3D-printed channels with glass covers. Immunostaining of nuclei (blue) and VE-cadherin (red) shows that on day 3 of the cell culture, the cells have fully developed adherens junctions among the cells. Phalloidin staining of cellular actin fibers (green) shows that those fibers, as well as the cell’s bodies, have aligned with the direction of fluidic flow (Figure 6D,E). On day 3, the alignment of cell bodies follows a Gaussian distribution with a standard deviation of 26°, compared to a standard deviation of 24° on day 2 and a standard deviation of 83° on day 1 of the cell culture. Those results are comparable to other fluidic endothelial cell cultures where the cells adhere to a glass surface, and medium flows through a 3D-printed channel or PDMS channels [41,42], indicating that the devices create conditions that are suitable for culturing endothelial cells under unidirectional fluidic flow.

Figure 6C shows HUVECs cultured within the collagen channel of device design 2 on day 3 of the cell culture. The cells were immunostained for VE-cadherin (red), actin (green), and nuclei (blue). Microscopy images show that the adherens junctions are well-developed on day 3, and that cellular alignment in the direction of fluidic flow took place (Figure 6F). Cell alignment angles on day 3 follow a Gaussian distribution with a standard deviation of 30°. The results are comparable to HUVEC cultures in other collagen-based channels that were exposed to unidirectional fluidic flow [43,44]. Cell viability remained consistently high, with no statistically significant change between day 1 (97.6 ± 2.9%) and day 3 (95.1 ± 2.9%) (*p* > 0.05), confirming robust cell survival during device operation.

### 3.5. Barrier Function

**Figure 7:**
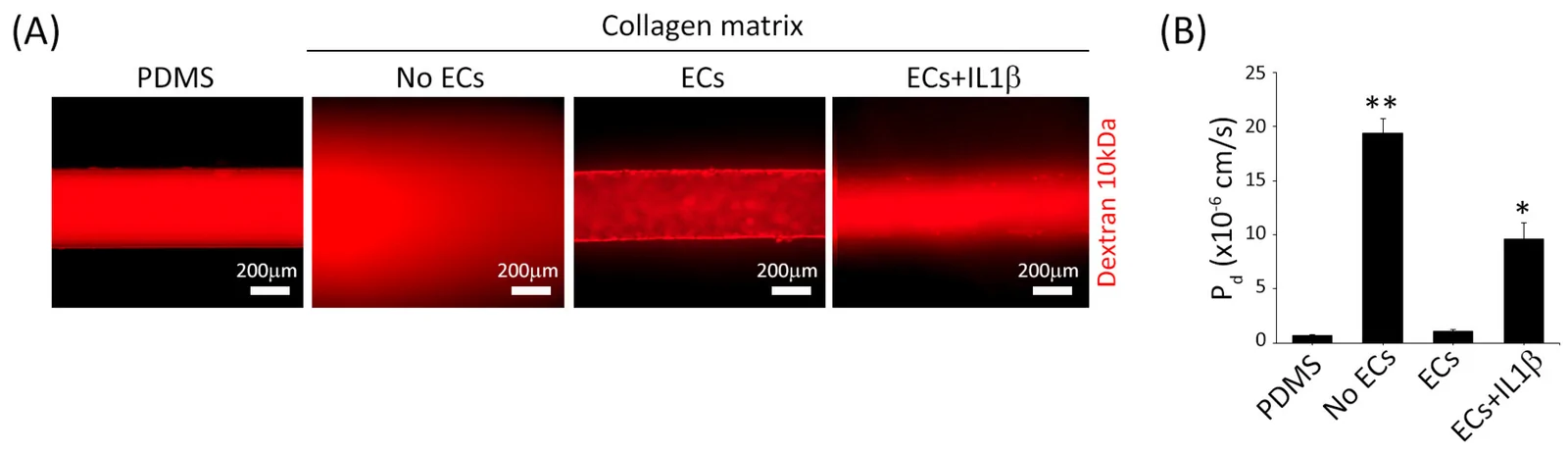
Endothelial cell barrier function using fluorescent dextran. (**A**) Representative images showing endothelial integrity using fluorescence-labelled 10 kDa dextran. (**B**) Graph demonstrated the endothelial leakiness measured by diffusive permeability coefficient (P_d_). Error bars represent ± the standard deviation of *n* = 3 to *n* = 6 measurements. * *p* < 0.05 ECs vs. ECs + IL1β; ** *p* < 0.01 ECs vs. No ECs.

To characterize the barrier function of the in vitro endothelial lining we created, we added fluorescently labeled dextrans, 10 kDa in size, to the cell culture medium. We recorded the diffusion of those dextrans and measured the fluorescence outside of the channel within a pre-specified region of interest. On day 3 of the cell culture, we observed that the cells had developed excellent barrier functionality. We observed no significant difference in fluorescence in the region of interest between cell-lined collagen channels (1.1 ± 0.2) (*p* < 0.01) and PDMS control channels that are impermeable to 10 kDa dextran (0.7 ± 0.1 for 10 kDa dextran) (Figure 7). When inflammatory proteins (IL1β) were added to the cell-lined collagen channels, fluorescence in the collagen matrix increased approximately 10-fold to 9.6 ± 1.5 (*p* < 0.05) compared to the control without inflammatory protein addition (Figure 7).

### 3.6. System Design

Using 3D printing for rapid prototyping of our devices, we were able to realize the most important design criteria needed to achieve mostly unidirectional fluidic flow. Those criteria are (1) that the fluidic cell culture channel connecting the two reservoirs, as well as any connecting channel segments, are all small enough to hold cell culture medium within them via capillary forces when either reservoir is empty; (2) that the cell culture medium inside the medium reservoir completely disconnects from the medium inside the microfluidic channels when the device is rotated or tilted; and (3) that the cell culture medium inside the u-shaped reservoir reconnects with the medium inside the microfluidic channels once the rotation or tilting motion is completed.

The overall design was adaptable to two configurations, one with a plastic/glass channel (device 1), and one with a collagen channel (device 2). The design that accommodated the collagen channel needed to be slightly larger than the design with the plastic/glass channel to accommodate the microfluidic channel, but the three design criteria were maintained. This design also required an additional access hole for the syringe that served as a channel molding master for the collagen channel. This access hole was closed upon completion of the channel using tape. While the design of both versions of the device looked slightly different, the cell-seeding protocol and the cell culture protocol were the same for both.

## 4. Discussion

### 4.1. System Design, Fabrication, and Operation

Our goal for this study was to develop a pumpless microfluidic device that is versatile, easy to use, and that can accommodate many biomedical applications. The design criteria we developed and tested are simple, and we demonstrate their application with two device designs. Using those criteria, we believe that many other variations are possible. The criteria we developed are for (1) when the device is in motion, the cell culture medium in the medium reservoirs must completely disconnect from the medium inside the microfluidic channel; (2) when the device is in motion and the medium is disconnected, the fluidic connections between the cell culture channel and the medium reservoirs must be small enough to retain cell culture medium inside the cell culture channel via capillary forces; and (3) once the device is no longer in motion, cell culture medium in the upper inlet, must seamlessly reconnect with the cell culture medium in the microfluidic cell culture channel without generating an air bubble.

Here, we used 3D printing to create prototype devices of our designs and determined that 3D printing is a suitable but not necessarily ideal fabrication method. When using 3D printing to construct the devices, we were largely able to achieve our design criteria. However, 3D printers often construct devices layer by layer. In our case, we printed with a layer height of 100 µm. When multiple layers of material are used to create a rounded surface such as that of the round medium reservoirs, the surface will contain microscale ridges on the order of the layer height. Those microscale ridges can present capillary forces that act on the cell culture medium and prevent the complete disconnection of fluid from the microfluidic channel. When such capillary forces are present, the fluidic flow in the devices will likely result in brief periods of reverse flow, as seen in Figure 3 for 254 µm and 508 µm channels and in Figure 4 for 508 µm and 762 µm channels. To clarify, this transient behavior produces a mostly unidirectional flow, where the net fluid transport is unidirectional despite brief reverse-flow moments during rocking platform transitions. To achieve completely unidirectional flow without these brief reverse periods, all internal device surfaces should be as smooth as possible. Using higher-resolution printers or other fabrication methods that deliver smoother prints is best suited to achieve that.

While experimenting, we also observed that reverse flow can occur when the devices are operated with excess cell culture medium. While optimizing our operation parameters, we observed that a moment of reverse flow can occur when the top reservoir contains excess liquid when the device moves because the disconnection of the liquid in the reservoir from that in the channel is delayed. This condition can be rectified by simply decreasing the amount of medium in the system or by lengthening the time between device movements.

### 4.2. Flow Rates

It is possible to utilize our design criteria to create devices that deliver fluidic flow rates for almost any application. Variables that affect the flow rate inside the cell culture channel are (1) the size of that channel, (2) the geometry of the medium reservoir, and (3) the operational parameters.

(1) The microfluidic channel dimensions can be adjusted to tune pressure drops and the corresponding flow rates (Equation (2)). Both channel length and diameter influence the pressure drop, with the diameter exerting a stronger effect. Longer microfluidic channels and larger cell culture chambers can be accommodated by also elongating the medium reservoir. Elongating that reservoir can also accommodate larger amounts of cell culture medium, should that be needed;
(2) The size and shape of the medium reservoir can also be changed. Here, we designed a c-shaped reservoir and a u-shaped reservoir. Here, it is important to keep in mind that larger reservoir diameters will reduce forces due to surface tension at the reservoir wall. Those surface tensions are responsible for shaping the flow rates in addition to the pressure drop between the medium reservoirs and the hydraulic resistances of the microfluidic channels discussed above. If needed, the surface tension at the reservoir walls can also be changed by changing the cell culture medium contact angle of the device material via coating with hydrophobic or hydrophilic coatings. However, device coatings must be characterized with regard to their substance absorption, in case substances or drugs are involved in the study, since such absorption can considerably change the availability of the substance in the cell culture medium;
(3) The angle at which the rotating platform is operated can significantly affect the flow rate. Here, our custom-made rotating platform has advantages over typical rocking platforms. The angle at which the devices are tilted can be adjusted from 1°, resulting in lowered pressure drops between the medium reservoirs, to 90°, resulting in the maximum possible pressure drop for any particular device design (Figure 2). Typical rocking platforms maintain platform angles of 15° to 20°. Here, to achieve higher flowrates, we custom-modified the platform angle of the rocker platform to achieve 45°, but this may not always be possible. It should be noted that the reported wall shear stresses were calculated based on volumetric flow measurements from external sensors and analytical fluid mechanics models, rather than direct intra-channel flow visualization (e.g., micro-particle image velocimetry, PIV or Computational Fluid Dynamics, CFD) [45,46]. Although direct local velocity profiling was not performed, the robust cellular alignment along the flow direction (Figure 6) and the establishment of functional endothelial barrier integrity (Figure 7) provides strong biological validation that physiologically relevant wall shear stress was successfully transmitted to the cultured cells. Looking forward, incorporating detailed CFD simulations and micro-PIV will be important to refine local shear stress distributions within complex channels. Furthermore, future work could integrate fractional dynamics to model complex cellular responses under dynamic flow [47].

### 4.3. Design Limits

Despite the design flexibility that our design criteria offer, there is a lower size limit that must be kept in mind. When the medium reservoirs become small in diameter (below about 5 mm), the movement of the medium creates air bubbles. The presence of air bubbles can disturb the process of the reservoir medium, disconnecting from the medium in the channel and, with that, interrupting the unidirectional flow pattern.

Our device was open to the environmental air to allow for gas exchange between the cell culture medium and the ambient air. While this does not present a problem for larger devices that are operated with several mL of cell culture medium, it can become a consideration for devices like ours that aim to operate with small amounts of recirculating medium. Even if only a small percentage of water evaporates, evaporation can have a concentrating effect on the cell culture medium, thereby also affecting the overall osmotic pressure acting on the cells. We found that evaporation can be effectively mitigated by reducing the access hole diameter (2 mm in our devices), using a loose-fitting cover (e.g., small petri dishes), and operating in a humidified environment. In addition, exchanging the cell culture medium often and washing out old medium (at least every 24 h) allowed us to reset the amount of medium inside the devices.

It should also be mentioned that creating gravity-driven fluidic flow with a rotating platform exposes the cells to a slightly different pattern of gravity that acts on the cells’ bodies during the culture. Although a more thorough analysis would be needed to draw any particular conclusions, we did not see any evidence in terms of cell viability or barrier function that this pattern of gravity was detrimental to the growth of HUVECs.

### 4.4. Biological Validation

We cultured HUVECs in two separate device designs that were based on the same design principles but resulted in slightly different device configurations, one where cells grew on a fibronectin-coated glass surface, and one where cells grew inside a collagen matrix channel. In both designs, HUVECs were viable for the three days we ran the experiments. We observed that, over the 48 h of culture, cells establish adherens junctions, as seen via immunostained cultures in Figure 6, but that, at that time point, the cells have not fully aligned to the direction of flow. After 72 h, the cells retain their adherens junctions, and a larger number of cells align to the direction of flow. Given those results, we estimate that experiments to determine transport rates of substances can be conducted on day 3 of the cell culture.

Our device design accommodated cell seeding through an access hole (2 mm in diameter) through which we pipetted the cell suspension. This hole also accommodates the necessary gas exchange between cell culture medium and ambient air. However, using this method for cell seeding, it can be hard to exactly control the cell-seeding density. A possible alternative design could feature two smaller, cone-shaped cell seeding ports at the beginning and at the end of the microfluidic channels. In the future, such a design might make cell seeding more reliable.

In line with recent advances in microfluidic organ-on-a-chip platforms, which emphasize the precise replication of physiological flow and barrier functions for biomimetic disease modeling [48,49,50], our recirculating system produces fluid shear stress that maintains endothelial barrier integrity and functional responsiveness.

To establish the functional performance of our microfluidic platform, we performed a multi-modal characterization suite combining structural imaging, viability assays, and solute transport measurements. Continuous localization of VE-cadherin, flow-aligned F-actin microfilaments, and FITC-dextran permeability assays confirmed that the device maintains functional junctional assembly and barrier tightness under recirculating flow. Together with high cell viability (>95%), this evaluation provides biological validation that the device supports a healthy, physiologically responsive barrier tissue. Additional molecular markers—such as CD31/PECAM-1 for cell lineage verification [51,52] or eNOS for vasoactive signaling [53,54,55]—could be incorporated into future disease-modeling or pharmacological studies.

### 4.5. Advantages and Future Applications

To contextualize the performance and operational benefits of our system, we benchmarked our platform against existing gravity-driven and pumpless MPSs in terms of flow directionality, recirculating capability, operating volume, flow rate, shear stress, fabrication complexity, and scalability (Table 1). The system we developed is versatile with regard to the flow and shear rates it can be operated with. The design and operating parameters of the devices are suitable for operating single-organ cell cultures where either low shear for non-barrier tissues or higher shear for barrier tissues is required. Here, we demonstrate the devices’ operation with a single channel and high shear conditions. Lowering the shear to values suitable to culture tissues that in vivo only experience the flow of interstitial fluid can be achieved by using one of the methods discussed above. Both rocker and rotating platforms can be used to achieve low, medium, and high flow rates.

Our devices can accommodate extracellular matrix to generate complex, perfused 3D models of tissues. To build such tissues, it may be possible in future studies to embed multiple cell types in the extracellular matrix. The channels can then be lined with HUVEC, as demonstrated here, or with tissue-specific endothelial cells. This approach holds the potential to mimic and observe more complex tissue structures and create more in vivo-like inter-cellular interactions between a tissue and its vasculature. For example, future configurations could aim to simulate the interactions of ECs with collagen-embedded hepatocytes [23,56] under unidirectional and recirculating fluidic flow.

A major advantage of our device is that it has very small dead volumes and can recirculate very small amounts of cell culture medium. The amount of medium needed to operate the devices is only limited by the rate of evaporation that occurs during operation. Because of the small amounts of medium needed, our devices enable growth factors, or any proteins excreted by the cell culture, to stay within the system and in circulation. In a system that requires more liquid, those factors would be diluted, and, in a single-pass system, such factors would even be lost. In future applications, the possibility to recirculate cell culture medium may facilitate experimentation with rare and expensive cells such as patient-derived circulating tumor cells (CTC), CAR-T cells, or other experimental chemical compounds.

Overall, the design criteria developed here could potentially be extended to create new designs that accommodate more complex fluidic networks, perhaps encompassing passive flow splitters (as previously discussed [19]) to explore multi-channel configurations or multi-organ platforms that contain cell culture chambers for several in vitro organ mimics [57,58], enabling inter-organ systems, where interactions among organs can be generated and examined.

While this study focuses on the primary engineering validation and short-term (72 h) characterization of HUVEC monolayers, the platform provides a foundation for broader applications. Notably, the functional responsiveness of the barrier was confirmed via IL1β stimulation, which induced measurable barrier disruption and increased dextran permeability (Figure 7). Future studies can leverage this recirculating architecture to evaluate long-term tissue maturation, integrate complex co-cultures incorporating epithelial or stromal cell lines, and complement solute transport assays [59] with real-time transendothelial electrical resistance (TEER) measurements [60].

## 5. Conclusions

Based on three design principles, we have created two versions of pumpless microfluidic cell culture devices that can be operated using gravity to generate unidirectional, recirculating fluidic flow of small amounts of cell culture medium. We have demonstrated that operation on two platforms, rocking and rotating, can create unidirectional flow patterns and rates of flow that are suitable for the culture of both barrier tissues and non-barrier tissues, such as tissues that require very low shear and tissues that thrive under high shear conditions. The highest achieved wall shear values were suitable to culture endothelial cells, which we demonstrated with the culture of HUVEC. While our analytical fluid mechanics model predicts flow rates for standard channel geometries, its predictions are subject to boundary limitations in a larger channel. We envision that the design principles demonstrated here could be adapted in the future for the culture of iPSC-derived endothelial cells, barrier tissues such as the endothelium, the GI tract epithelium, or the kidney epithelium. Because small amounts of cell culture medium can be recirculated, it is possible to retain any growth factors that the tissue generates within circulation. Future studies could also explore the feasibility of testing therapeutic cells or patient-derived cells within this platform.

## Figures and Tables

**Table 1:** Comparison of gravity-driven MPS platforms.

Performance Parameter	Traditional Rocking MPS ^a^	UniChip/Passive Route ^b^	3D-Tilting rOoC ^c^	This Work
Flow Directionality	Bidirectional	Unidirectional	Unidirectional	Unidirectional
Recirculating Capability	No (Bidirectional flow)	Yes (Passive micro-valves)	Yes (3D-tilting platform)	Yes (High efficiency, >87–96%)
Operating Volume	High (1500–3000 μL)	Moderate (200–500 μL)	Moderate (>300 μL)	Low to High (100–5000 μL)
Achievable Flow Rate	Low to Moderate (5–34 μL/min)	Moderate to High (7–35 μL/min, 320–570 μL/min)	Physiological (20–150 μL/min)	Physiological (18–144 μL/min)
Wall Shear Stress	Low/Oscillating (<0.02 Pa)	Physiological (0.53 Pa)	Physiological (0.03–0.30 Pa)	Physiological (0.04–0.45 Pa)
Fabrication Complexity	Moderate (PDMS/PMMA Gasket)	High (Multi-layer Micro-valves)	Moderate to High (Requires 3D Stage)	Low (3D printing + Glass/ECM)

^a^ Sung et al. (2010), Miller et al. (2016) [18,28]; ^b^ Wang et al. (2018), LaValley et al. (2021) [16,24]; ^c^ Busek et al. (2023) [15].

## Data Availability

The data that support the findings of this study are available from the corresponding author upon reasonable request.
